# Glycine receptor in rat hippocampal and spinal cord neurons as a molecular target for rapid actions of 17-β-estradiol

**DOI:** 10.1186/1744-8069-5-2

**Published:** 2009-01-12

**Authors:** Peng Jiang, Yan Kong, Xiao-Bing Zhang, Wei Wang, Chun-Feng Liu, Tian-Le Xu

**Affiliations:** 1 Institute of Neuroscience and State Key Laboratory of Neuroscience, Chinese Academy of Sciences, Shanghai 200031, PR China; 2 Department of Neurology, Second Affiliated Hospital of Soochow University, Suzhou 215004, PR China

## Abstract

Glycine receptors (GlyRs) play important roles in regulating hippocampal neural network activity and spinal nociception. Here we show that, in cultured rat hippocampal (HIP) and spinal dorsal horn (SDH) neurons, 17-β-estradiol (E_2_) rapidly and reversibly reduced the peak amplitude of whole-cell glycine-activated currents (*I*_Gly_). In outside-out membrane patches from HIP neurons devoid of nuclei, E_2 _similarly inhibited *I*_Gly_, suggesting a non-genomic characteristic. Moreover, the E_2 _effect on *I*_Gly _persisted in the presence of the calcium chelator BAPTA, the protein kinase inhibitor staurosporine, the classical ER (i.e. ERα and ERβ) antagonist tamoxifen, or the G-protein modulators, favoring a direct action of E_2 _on GlyRs. In HEK293 cells expressing various combinations of GlyR subunits, E_2 _only affected the *I*_Gly _in cells expressing α2, α2β or α3β subunits, suggesting that either α2-containing or α3β-GlyRs mediate the E_2 _effect observed in neurons. Furthermore, E_2 _inhibited the GlyR-mediated tonic current in pyramidal neurons of HIP CA1 region, where abundant GlyR α2 subunit is expressed. We suggest that the neuronal GlyR is a novel molecular target of E_2 _which directly inhibits the function of GlyRs in the HIP and SDH regions. This finding may shed new light on premenstrual dysphoric disorder and the gender differences in pain sensation at the CNS level.

## Background

Studies over the last several decades have demonstrated that estrogen plays an important role in not only reproduction, but also regulation of early CNS development [1] and in synaptic plasticity of the mature hippocampus [2]. The classical estrogen actions in the CNS are primarily mediated by activating nuclear estrogen receptor α and β (ERα/β), causing long-term genomic effects [3,4]. Nevertheless, it is becoming increasingly clear that estrogen can activate cytoplasmic signaling events at or near the plasma membrane [5,6], presumably through either membrane-localized classical ERs [7,8] or novel ERs [9-11]. Moreover, estradiol is reported to directly bind to and modulate certain ion channels, like the Maxi-K channels [12], indicating the existence of additional estrogen targets besides ERs. In the hippocampus, both *in vivo *[13] and *in vitro *[14-16] studies have focused on the inhibitory GABAergic machineries, and suggested that estradiol alters neuronal activity by suppressing GABAergic synaptic transmission. A recent study also indicated that estradiol inhibits human recombinant rho1 GABA_C _receptor [17].

Like GABA_A _receptors (GABA_A_Rs), the major receptor mediating central inhibition, GlyRs contribute to neuronal inhibition in hippocampus [18-20] and spinal cord [21,22]. GlyRs are pentamers and composed of α(1–4) and β subunits [21]. In hippocampal (HIP) neurons, GlyRs are thought to be primarily the homopentamer of α2 subunits that function extrasynaptically to produce tonic inhibition [21]. Tonic activation of GlyRs leads to cross-inhibition of GABA_A_Rs [23], and influences synaptic activity [18,24,25] and short-term plasticity [19]. In adult spinal dorsal horn (SDH), GlyRs are important in regulating nociception and motor function. For example, α3-containing GlyRs regulate inflammatory pain sensitization [26]. Interestingly, during the development of the spinal cord, there is a switch of GlyR subunit composition from α2 in the fetus to α1 predominance in the adult [21,27], suggesting a role of the α2 subunit in neuronal development. Indeed, two recent studies showed that GlyRs play an important role in rod photoreceptor development of the vertebrate retina [28] and regulate spinal interneuron differentiation in zebrafish [29]. On the other hand, estrogen is locally synthesized in the CNS [30] and the level of estrogen is under regulation [1,31]. A recent study showed that estradiol enhances the spontaneous synaptic release of glycine in hypoglossal motoneurones [32]. However, the estradiol effects on GlyRs remain unexplored. In this study, therefore, we examined the modulatory effects of 17-β-estradiol (E_2_), the most prevalent and potent form of endogenous estrogen, on native GlyRs in HIP and SDH neurons, and on recombinant GlyRs expressed in HEK293 cells. This study will add a new dimension for understanding the multifaceted estrogenic effects in the CNS.

## Results

### 17-β-estradiol rapidly inhibits glycine-activated current (I_Gly_) in cultured rat SDH and HIP neurons

**Figure 1:**
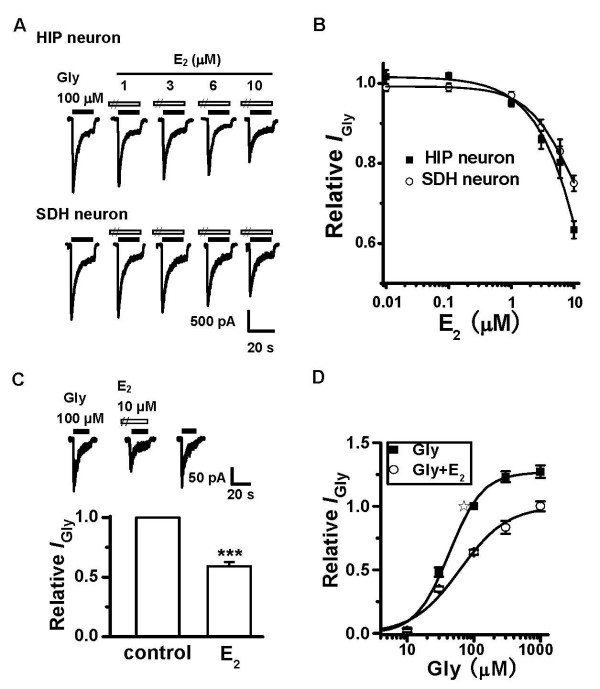
**E_2_-induced inhibition of *I*_Gly _in cultured SDH and HIP neurons**. (A) Representative traces of current induced by 100 μM glycine in the presence or absence of E_2 _at various concentrations in cultured HIP (upper) and SDH (bottom) neurons. The neurons were pre-treated with E_2 _for 30 s before E_2 _and glycine were co-applied. (B) Summarized data illustrating the concentration dependence of E_2 _inhibition (n = 4–8) as shown in *A*. (C) E_2 _significantly inhibited *I*_Gly _recorded from the outside-out patches (n = 5). The upper traces show the representative *I*_Gly _recorded from outside-out patches in the presence and absence of E_2_. ***P < 0.001, Paired Student's *t*-test, compared with control without adding E_2_. (D) The concentration-response curves of *I*_Gly _in the presence and absence of 10 μM E_2_. For each neuron recorded, the current was normalized to the peak amplitude of *I*_Gly _induced by 100 μM glycine alone (✰) from the same neuron and each point represents the average value of 5–9 neurons.

At a holding potential (*V*_H_) of -50 mV under whole-cell voltage clamp, application of glycine (100 μM) to the cultured HIP or SDH neurons elicited an inward current. The strychnine sensitivity and chloride dependence of the *I*_Gly _suggests that it was mediated by GlyR-chloride channels (data not shown). After recording a stable control *I*_Gly_, we pre-superfused the neurons with E_2 _at various concentrations for 30 s, and then recorded *I*_Gly _in the presence of E_2_. The peak amplitude of *I*_Gly _was rapidly reduced by E_2 _application (Figure 1A), while it was not further inhibited when the pre-perfusion time was prolonged (data not shown). In neurons derived from neonatal rats of both sexes, E_2 _exerted a similar inhibitory effect on *I*_Gly_, therefore data from both sexes were pooled for comparison. As shown in Figure 1B, E_2 _concentration-dependently inhibited the peak *I*_Gly_. On average, E_2 _at 1, 3, 6 and 10 μM significantly reduced peak *I*_Gly _to 95.0 ± 0.8% (*P *< 0.05 compared with control, n = 4), 85.8 ± 2.2% (*P *< 0.01 compared with control, n = 5), 80.2 ± 4.0% (*P *< 0.01 compared with control, n = 5) and 63.4 ± 2.2% (*P *< 0.001 compared with control, n = 8, Paired Student's *t*-test for all) of control in HIP neurons, respectively; and in SDH neurons, peak *I*_Gly _was reduced to 97.0 ± 0.9% (*P *< 0.05 compared with control, n = 6), 89.1 ± 2.0% (*P *< 0.01 compared with control, n = 7), 83.1 ± 3.0% (*P *< 0.01 compared with control, n = 8) and 75.3 ± 2.1% (*P *< 0.01 compared with control, n = 7, *P *< 0.01 compared with the inhibition of 10 μM E_2 _produced in HIP neuron) of control, respectively. The IC_50 _values of E_2 _for *I*_Gly _of the HIP neuron and SDH neuron are 16.5 ± 2.7 μM and 33.2 ± 3.4 μM (*P *< 0.01 compared with that in HIP neuron), respectively. Therefore E_2 _has stronger inhibitory effect on *I*_Gly _mediated by hippocampal GlyR. To assess possible contaminations of endogenous steroids derived from glial cells [30,33], we examined the E_2 _effect in cultures grown without blocking the proliferation of glial cells and found that E_2 _still inhibited *I*_Gly _under such a condition (data not shown).

The concentration-effect relationships for E_2 _shown in Figure 1B indicate that E_2 _inhibited *I*_Gly _more markedly in cultured HIP neurons. In the following experiments, therefore, we used HIP neurons to study the mechanisms underlying E_2 _inhibition. To explore whether a genomic mechanism is responsible for E_2 _inhibition on *I*_Gly_, we first examined the effect of E_2 _in large outside-out patches excised from cultured HIP neurons. The patches were exposed to rapid changes of glycine or glycine plus E_2_. As shown in Figure 1C, the peak amplitude of *I*_Gly _was significantly inhibited by E_2 _(59.2 ± 3.6% of control, *P *< 0.001, Paired Student's *t*-test). Since outside-out patches contain no cellular nuclei, this result indicates that E_2 _inhibited *I*_Gly _via a non-genomic mechanism. We next examined the concentration-response relationships of *I*_Gly _in the absence or presence of E_2_. As shown in Figure 1D, E_2 _at 10 μM suppressed *I*_Gly _evoked by both subsaturating and saturating concentrations of glycine. The EC_50 _and Hill coefficient of glycine were 42.9 ± 4.3 μM and 1.7 in the absence of E_2 _and 61.7 ± 8.1 μM and 1.2 in the presence of E_2_, respectively. This result suggests that E_2 _inhibits *I*_Gly _in a noncompetitive manner.

### No involvement of intracellular signaling pathways and classical ERs

**Figure 2:**
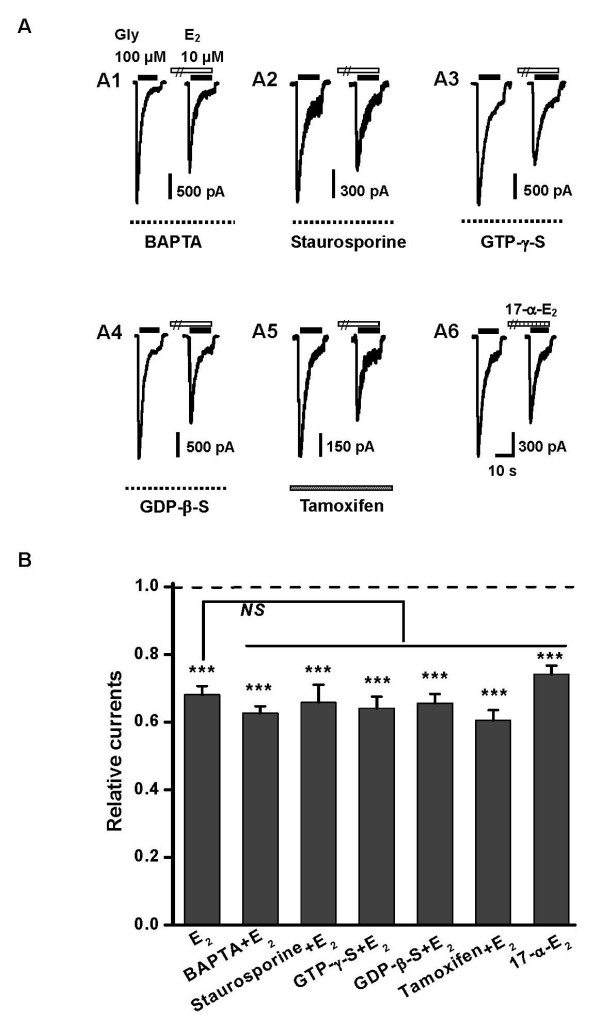
**E_2_-induced inhibition of *I*_Gly _is independent of intracellular signaling pathways and classical estrogen receptors (ERs)**. (A) Sample traces illustrating the inhibitory effects of E_2 _on the peak *I*_Gly _under the conditions of intracellular application 15 mM BAPTA (A1), 5 μM staurosporine (A2), 0.5 mM GTP-γ-S (A3) and 0.5 mM GTP-β-S (A4), respectively. A5, Effect of E_2 _on *I*_Gly _after incubation of neurons with tamoxifen for 2 h. A6, Effect of 17-α-E_2 _on *I*_Gly_. (B) Pooled data summarizing the effect of E_2 _on *I*_Gly _under various conditions shown in *A*. Each column represents the average values from 4–6 neurons, ***P < 0.001, Paired Student's *t*-test, compared with control without adding E_2 _or 17-α-E_2 _(dashed line). *NS *indicates no significant difference in this and the following figures.

Previous studies have indicated that the acute effect of E_2 _occurring within a time course of milliseconds to minutes are attributed to the activation of intracellular signaling pathways mediated by presumably membrane-bound classical ERs [7,8,34,35] or novel ERs [9,10]. Additionally, E_2 _can modulate calcium channels and affect the intracellular calcium level [36,37]. To explore the possible involvement of any intracellular pathways in mediating E_2 _inhibition of *I*_Gly_, following experiments were conducted. We first examined the role of the intracellular Ca^2+^. When neurons were loaded with 15 mM BAPTA via the recording pipette, E_2 _reduced the peak *I*_Gly _to 62.7 ± 2.0% of the control, which was not significantly different from that obtained in the absence of BAPTA (Figure 2A1 and 2B1, *P *> 0.05, Unpaired Student's *t*-test). In order to test the role of protein phosphorylation and dephosphorylation in E_2 _inhibition, we loaded the neurons with staurosporine (5 μM), a nonselective protein kinase inhibitor, to disrupt the balance between phosphorylation and dephosphorylation. Likewise, the inhibitory effect of E_2 _on *I*_Gly _was not altered (Figure 2A2 and 2C1, *P *> 0.05, Unpaired Student's *t*-test). A previous study [38] demonstrated that the GlyR is a target of the G protein βγ dimer. To examine the role of G proteins, we loaded the neurons with GTP-γ-S (500 μM) or GDP-β-S (500 μM) to activate or block the G protein, respectively. Neither of these treatments affected the inhibition induced by E_2 _on *I*_Gly _(Figure 2A3, A4, and 2C, *P *> 0.05, Unpaired Student's *t*-test). Thus, it is unlikely that E_2 _exerts its inhibition on *I*_Gly _through intracellular signaling pathways.

We further employed tamoxifen, a classical ER antagonist in the hippocampus [15], to examine whether membrane-localized classical ERs were involved in E_2 _inhibition of *I*_Gly_. After incubation of 1 μM tamoxifen for 2 h, we examined the effects of E_2_on *I*_Gly _in the continued presence of tamoxifen. Though, consistent with the previous study [32], the peak amplitude of *I*_Gly _induced by 100 μM glycine was decreased after the treatment with tamoxifen (Figure 2A5, note the difference in the scale bar), the inhibitory effect of E_2 _on *I*_Gly _was not affected (Figure 4A5 and 4B1, *P *> 0.05, Unpaired Student's *t*-test). Moreover, 17-α-estradiol (17-α-E_2_, 10 μM), the inactive stereoisomer of E_2_, mimicked the inhibitory effect of E_2 _on *I*_Gly _(Figure 2A6 and 2B1, *P *> 0.05, Unpaired Student's *t*-test), suggesting that E_2 _inhibition on GlyR is independent of classical ERs.

### Regulatory sites for E_2 _and pregnanolone on GlyRs are separate

**Figure 3:**
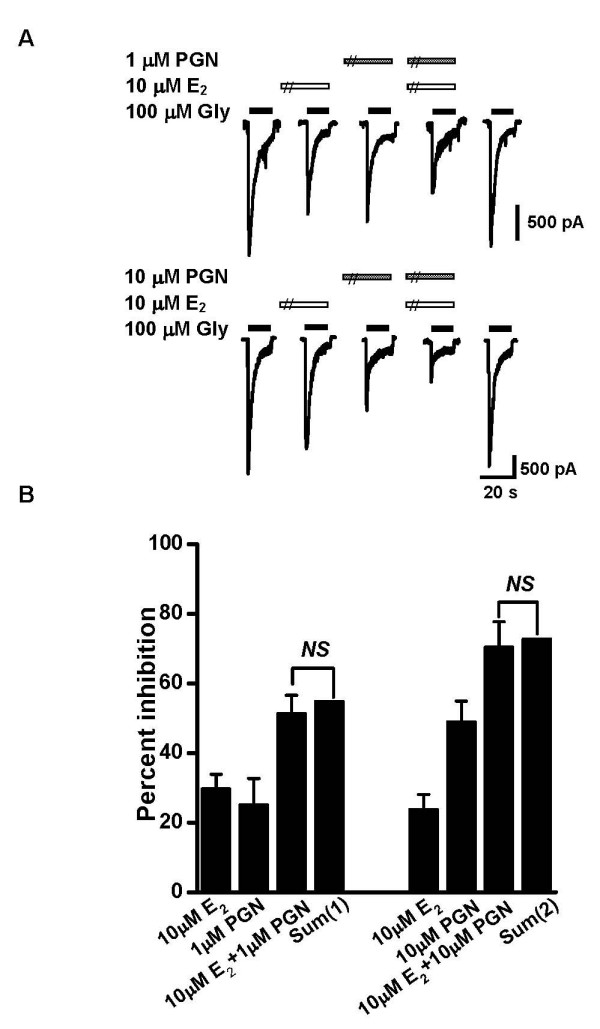
**Interactions of E_2 _and PGN on *I*_Gly_**. (A) Sample traces illustrating the additive effect of E_2 _and PGN on *I*_Gly_. (B) Summary of results from all experiments similar to those shown in *A *(n = 4–5). Sum (1) is the expected linear summation of the inhibition induced by 10 μM E_2 _and 1 μM PGN; Sum (2) is the expected linear summation of the inhibition induced by 10 μM E_2 _and 10 μM PGN. *P *> 0.05, Unpaired Student's *t*-test.

A previous study showed that another neurosteroid, pregnanolone (PGN) directly inhibited *I*_Gly _in a competitive manner [39]. We were interested to know whether E_2 _and PGN share a common binding site on GlyRs. If the sites are separate, the inhibitory effects should be additive when E_2 _and PGN were co-applied. As shown in Figure 3A, PGN at 1 μM and 10 μM significantly inhibited the peak amplitude of *I*_Gly _by 25.1 ± 7.6% and 49.0 ± 5.9% of control, respectively. In the presence of 10 μM E_2_, additional inhibition of *I*_Gly _was observed by PGN at both 1 μM (Figure 3B, 51.3 ± 5.3% of control), and 10 μM (Figure 3B, 70.4 ± 7.3% of control). These data suggest that distinct binding sites may mediate the inhibition of E_2 _and PGN on *I*_Gly_.

### Subunit selectivity of E_2 _inhibition on GlyRs

**Figure 4:**
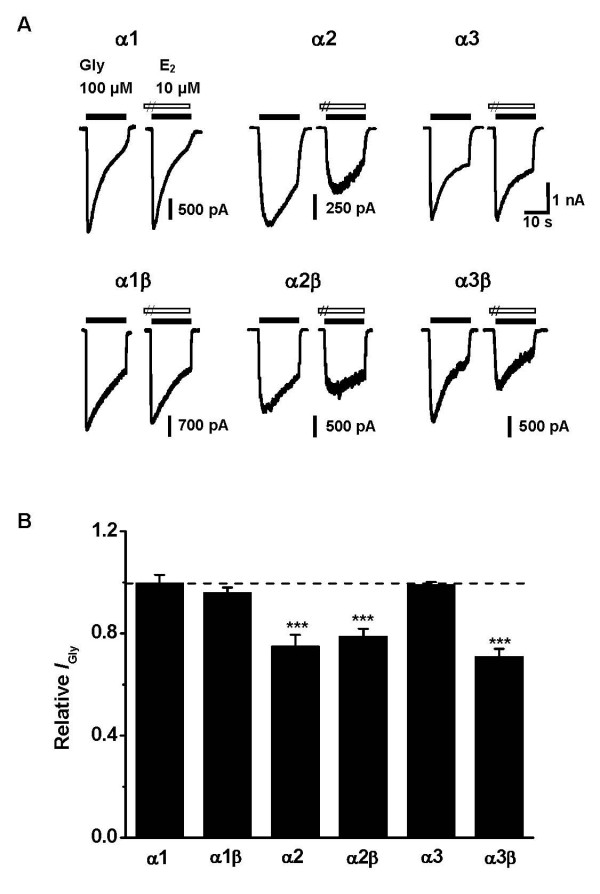
**Inhibitory effect of E_2 _on recombinant GlyRs**. (A) Sample traces demonstrating the effects of 10 μM E_2 _on various homomeric and heteromeric GlyRs. (B) Summary of results from all experiments similar to those shown in *A*. E_2 _selectively inhibited the peak amplitude of *I*_Gly _mediated by α2-containing GlyRs and α3β heteromeric GlyR. Each column represents the average value of 6–11 neurons. ***P < 0.001, Paired Student's *t*-test, compared with the control without E_2 _treatment (dashed line).

To determine which GlyR subunits are responsible for the E_2_-induced inhibition, we investigated the E_2 _effects on *I*_Gly _in HEK293 cells expressing various recombinant GlyRs (Figure 4A). In GlyR subunit-untransfected cells, no glycine responses were observed (data not shown). As shown in Figure 4, E_2 _selectively inhibited the peak *I*_Gly _mediated by homomeric α2-GlyRs to 75.1 ± 4.3% of control (*P *< 0.001, n = 6, Paired Student's *t*-test), but had no significant effect on homomeric α1- and α3-GlyRs (99.8 ± 3.1% of control and 99.0 ± 1.2% of control for α1- and α3-GlyRs, respectively, *P *> 0.05, n = 10, Paired Student's *t*-test). To further examine whether co-expression of β subunit affected the E_2 _inhibition, we tested the inhibitory effect of E_2 _on heteromric GlyRs. We found that E_2 _inhibited the peak *I*_Gly _mediated by α2β – and α3β-GlyRs to 78.9 ± 2.9% (*P *< 0.001, n = 9, Paired Student's *t*-test) and 71.0 ± 3.0% of control (*P *< 0.001, n = 11, Paired Student's *t*-test), respectively. However, *I*_Gly _mediated by α1β-GlyRs was not significantly affected. Thus, it is likely that either α2- or α2β – or α3β-GlyRs mediate the E_2 _effect in SDH and HIP neurons.

### The developmental changes of E_2 _inhibition of GlyRs in spinal cord neurons

**Figure 5:**
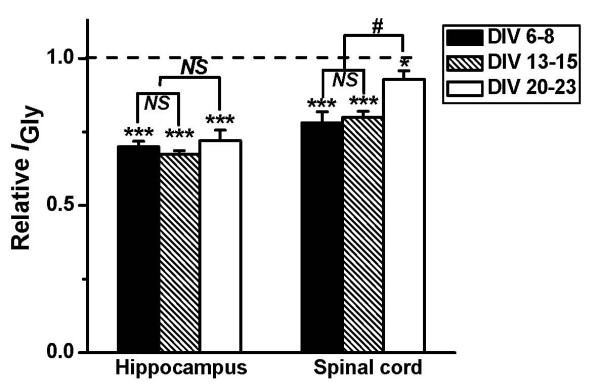
**Developmental regulation of E_2 _inhibition on *I*_Gly_**. Hippocampal and spinal cord neurons in culture were used for electrophysiological recording following 6–8, 13–15 and 20–23 days of *in vitro *(DIV) differentiation to examine the developmental dependence of E_2 _inhibition on *I*_Gly_. Each column represents the average value of 5–8 neurons. * *P *< 0.05 and *** *P *< 0.001 (Student's paired *t *test, n = 8), compared to control without adding E_2_; # *P *< 0.05 (one-way ANOVA, n = 8), comparing DIV 20–23 with either DIV 6–8 or DIV 13–15.

The GlyR subunit expression is developmentally regulated in some areas of CNS such as spinal cord. To test whether E_2 _effects are different in different times in culture, we investigate the effects of E_2 _on GlyR in spinal cord and HIP neurons from different days in culture. We first recorded *I*_Gly _in cultured HIP neurons after different days of *in vitro *(DIV) differentiation. As shown in Figure 5, the inhibitory extent of E_2 _on *I*_Gly _did not change with time *in vitro *(*P *> 0.05, comparing DIV6–8, 13–15 and 20–23, n = 5–7, one-way ANOVA). We next examined the effects of E_2 _on *I*_Gly _induced by 100 μM glycine of spinal cord neurons in cultures after DIV6–8, 13–15 and 20–23. Interestingly, E_2 _significantly inhibited *I*_Gly _of the neurons at DIV6–8 and 13–15, but the inhibitory effects declined in the neurons at DIV20–23 (*P *= 0.012, comparing between DIV20–23 and DIV6–8; *P *= 0.023, comparing between DIV20–23 and DIV13–15. n = 6–8, one-way ANOVA).

### Inhibitory effect of E_2 _on GlyR-mediated tonic current in HIP slices

**Figure 6:**
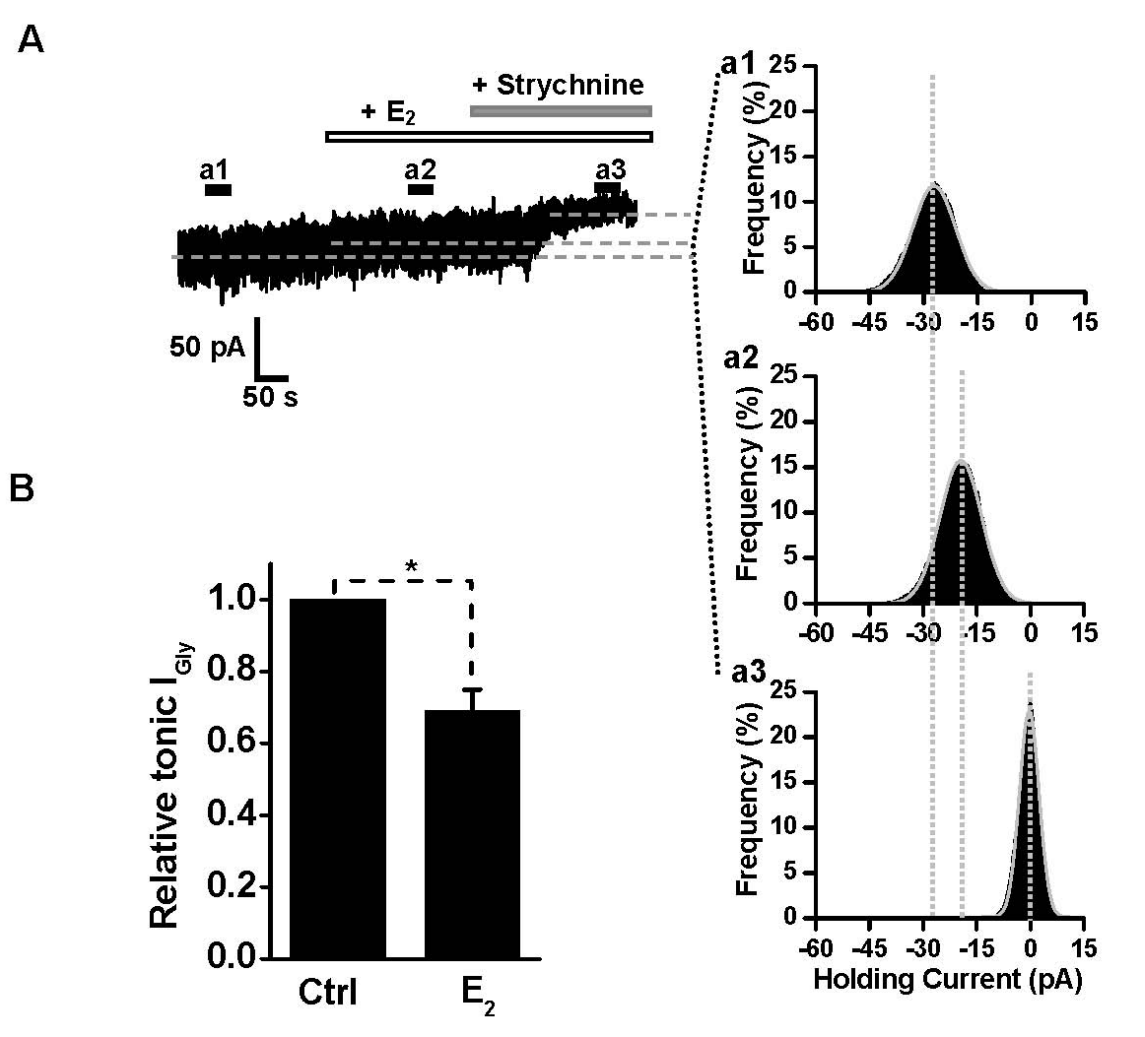
**Inhibitory effect of E_2 _on GlyR-mediated tonic current in HIP slices**. (A) Left, Whole-cell voltage-clamp recording showing the current in the presence of 0.3 μM TTX, 10 μM bicuculline, 3 μM CNQX, 10 μM APV, 20 μM glycine and 0.5 mM sarcosine. Application of strychnine (2 μM) decreased the membrane current noise and revealed the tonically activated GlyR current. In the presence of E_2 _(10 μM), the amplitude of GlyR-mediated tonic current was significantly reduced. Right (a1–a3), Gaussian fit to all-point histograms of 30 s traces at the time point a1, a2 and a3 (A, inset). The differences among the Gaussian means are marked by the dotted lines. (B) The normalized GlyR-mediated tonic current in the absence or presence of E_2 _(n = 8). *P < 0.05, Paired Student's *t*-test, compared with the control without E_2 _treatment.

In the developing and mature hippocampus, α2 subunit represents the primary component of the functional GlyRs [21]. This unique property enables us to examine the action of E_2 _on GlyRs from pyramidal neurons in CA1 region of HIP slices. Glycine concentration in cerebrospinal fluid has been estimated to be in the micromolar range [40]. In order to magnify the current, we recorded the GlyR-mediated tonic current by adding 20 μM glycine to ACSF in addition to the presence of GlyT1 inhibitor sarcosine (0.5 mM) [25]. At the same time, TTX (0.3 μM) was added in the bath to reduce random baseline current fluctuations, and 10 μM bicuculline, 10 μM DL-2-amino-5-phosphovaleric acid (APV) and 3 μM 6-cyano-7-nitroquinoxaline-2,3-dione (CNQX) were added to block GABA_A _and ionotropic glutamate receptors, respectively. After recording a control period in the cocktail solution, E_2 _at 10 μM was superfused (Figure 6A). As shown in Figure 6B, the GlyR-mediated tonic current was significantly reduced to 69.3 ± 5.8% of control by E_2 _perfusion (*P *= 0.043, n = 8, Wilcoxon matched-pairs signed-ranks test), a level compatible with that obtained in the cultured neurons (Figure 1B) or HEK 293 cells expressing recombinant α2- and α2β-GlyRs (Figure 4B).

## Discussion

Here, we present evidence that in cultured HIP and SDH neurons, E_2 _directly inhibited GlyRs which are likely composed of α2 or α3 subunit. Furthermore, the GlyR-mediated tonic current in HIP slices was reduced by E_2_. Thus, in addition to the well-known signaling pathways of E_2 _via ERs, the GlyR may be an additional central target of E_2_. Since GlyRs are important regulators of spinal nociception [26,41,42], and affect HIP network activity [19,20], our findings may shed new light on the gender differences in pain sensation at the CNS level.

### Mechanisms underlying E_2 _inhibition of GlyRs

Estrogen exerts effects in the CNS primarily by activating ERs [3]. In this study, however, several lines of evidence suggest that E_2 _inhibited *I*_Gly _through a direct interaction with plasma membrane GlyRs. First, the effects of E_2 _are most likely non-genomic as the inhibition occurred within seconds following E_2 _application and persisted in large outside-out patches devoid of nuclei. Second, it is unlikely that E_2 _physically disrupts the plasma membrane via a nonspecific way as E_2 _only affected α2 subunit-containing and heteromeric α3β-GlyRs. Moreover, when E_2 _was applied, the membrane capacitance remained unchanged, indicating that the membrane structure was unaltered (see Materials and Methods). Third, E_2 _inhibitory effect persisted in the presence of tamoxifen, the antagonist of classical ERs [43], or 17-α-E_2_, the inactive stereoisomer of E_2 _[44]. In addition, E_2 _inhibited the recombinant GlyRs expressed in HEK293 cells which are devoid of classical ERs [45]. Finally, in the presence of the calcium chelator, the protein kinase inhibitor or G-protein modulators, the E_2_-induced inhibition remained unchanged. These results strongly support the notion that E_2 _inhibits GlyRs independent of any intracellular signaling pathways activated by either classical ERs or novel ERs [9-11].

Our data suggest that E_2 _inhibited *I*_Gly _in a noncompetitive manner because E_2 _reduced *I*_Gly _independent of glycine concentrations, and the effect can be additive with that of PGN, a competitive steroid inhibitor of GlyR without changing the membrane viscosity [39,46]. Two general mechanisms may underlie the noncompetitive inhibition of E_2 _on GlyR, open-channel blocker or allosteric modulation. Nevertheless, E_2 _is not charged at physiological pH and inhibited *I*_Gly _independent of membrane potentials (P.J. and T.L.X., unpublished data), which makes it unlikely that E_2 _acts as an open-channel blocker. Thus, we propose that E_2 _directly binds to and allosterically inhibits GlyRs.

### Functional implications

Generally, the physiological concentration of estradiol in plasma is in the nanomolar range [6]. In the present study, the concentrations at which E_2 _exerted significant effects on GlyRs were much higher than its physiological level. However, in the CNS of both male and female rats, estrogen can be *de novo *synthesized and accumulated locally [30,47], leading to E_2 _concentration in the cerebrospinal fluid far surpass those measured in plasma. For example, a previous study has demonstrated that the concentration of estradiol in the hippocampus of male rats is six-fold higher than that in plasma [48]. More interestingly, a recent study indicates that forebrain estradiol levels in zebra finches are also acutely increased during social interactions [49]. In addition, certain cofactors, like sexual hormone-binding proteins, can make E_2 _to achieve an effect even at low concentrations [50]. Therefore, it is reasonable to speculate that, under certain physiological or pathological condition, locally elevated endogenous estrogen may have an impact on GlyRs through the process revealed in this study.

In HEK293 cells expressing recombinant GlyRs, we found that E_2 _significantly inhibited α2-GlyRs. Accumulative evidence demonstrates that the α2-GlyRs distribute throughout the developing CNS and in the mature hippocampus [21,51,52]. In CA1 region of HIP slices, we observed that GlyR-mediated tonic currents, which are likely mediated by α2-GlyRs [51-53], were reduced to a level compatible with that measured in cultured neurons or HEK 293 cells expressing recombinant GlyRs. The tonic activation of the α2 subunit-containing GlyRs is functionally important in the development of the cortex [54], the retina [28] and the spinal cord [29]. Furthermore, previous studies have shown that GlyR subunit composition of spinal cord neurons changes during development from α2 to α1 predominance both in cultures and in *in vivo *conditions [27,55]. Because of this switch of GlyR subunit composition in the spinal cord during development, a previous study [56] suggested that the selective inhibition of progesterone on α2-GlyRs endows an important role of progesterone in the developing spinal cord. Since the levels of estrogen fluctuate in both males and females during brain development [31], E_2_-induced inhibition of GlyRs may contribute to the modulation of estrogen on the early CNS development. It remains unexplored whether GlyRs also participate in regulation of neuronal excitability, through E_2_-induced disinhibition in the mature hippocampus [14].

Under certain disease conditions, for example, premenstrual dysphoric disorder (PMDD), anxiety is enhanced when neuronal excitability is disturbed during the ovarian cycle [57]. A recent study reported that the periodic alterations of the GABA_A_R-mediated tonic inhibition, which resulted from the fluctuation of hormones in the hippocampus during estrous cycle, play a crucial role in altered seizure susceptibility and anxiety during PMDD [58]. Similar to the tonic GABAergic inhibition, GlyRs-mediated tonic currents modulate neuronal excitation and network function [18,19,24,25]. Our present data showed that E_2 _can inhibit more than 30% of the tonic *I*_Gly _in hippocampal slices. Therefore, the significant inhibition of *I*_Gly _produced by E_2 _may have strong effect on the neuronal excitability, suggesting an alternative cellular mechanism by which periodic sex hormone fluctuations affect CNS neuronal excitation and cause mood disorders. Considering the ubiquitous distribution profiles of GABA_A_Rs in the CNS and the hypnotic side-effect of GABA-mimic drugs, the present finding suggests new directions for treatment of PMDD by using GlyR-enhancing drugs and/or glycine reuptake inhibitors [25].

Besides, sex-related difference in pain perception has been revealed by previous studies which indicate that noxious stimuli are perceived as more painful by women than by men [13,43]. Although sex hormones have been shown to be involved in this difference, the underlying mechanisms are still unknown. Functional α3β-GlyRs exist in inhibitory synapse of SDH neurons, which is the first site for integration, relay and modulation of nociceptive information from nociceptor [59]. Moreover, previous studies have suggested that α3β-GlyRs in the SDH neurons regulate inflammatory pain sensitization [26,41]. In this study, we noted that E_2 _significantly inhibited *I*_Gly _in cultured SDH neurons and the current mediated by α3β-GlyRs expressed in HEK293 cells, providing important insights into the mechanisms underlying gender differences in pain sensation at the CNS level.

Finally, several studies indicate that the expression of high affinity GlyR subunit α2^192L ^and α3^185L ^produced by RNA editing [60] was increased in the brain after experimentally induced brain lesion of rat [61] and in temporal lobe epilepsy patient with a severe course of disease [62]. According to the selectivity of E_2 _inhibition on α2 and α3-containing GlyR, E_2 _thus may play a role through modulating these high affinity GlyRs in some pathological situations such as temporal lobe epilepsy and brain damage.

## Conclusion

We demonstrated that the neuronal GlyR is a novel molecular target of E_2 _which directly inhibits the function of GlyRs in the HIP and SDH regions. Through their impact on GlyRs in the CNS, sex hormones may regulate neuronal excitability and contribute to premenstrual dysphoric disorder and sex-related pain sensation under both physiological and pathological conditions.

## Methods

The care and use of animals in these experiments followed the guidelines of the Institutional Animals Care and Use Committee of the Institute of Neuroscience, Shanghai Institutes for Biological Sciences, Chinese Academy of Sciences. All efforts were made to minimize the number of animals used and their suffering.

### Cell culture

Cultures of HIP neurons were prepared as previously described [63] with some modifications. Briefly, hippocampus from neonatal (< 24-hour-old) Sprague-Dawley rats with identified sex were dissociated in Ca^2+^-free saline with sucrose (20 mM) and hippocampal neurons were isolated using a standard enzyme treatment protocol. Cultures of SDH neurons were prepared from embryonic day 15 (E15) Sprague-Dawley rats as previously described [39]. The neurons were plated (1–5 × 10^5 ^cell/ml) on poly-L-lysine (Sigma, USA) coated cover glasses and grown in Dulbecco's modified Eagle's medium (DMEM, Gibco, USA) with L-glutamine plus 10% fetal bovine serum (Gibco). The neurons were allowed to attach the cover glasses for 24 h, after which the medium was changed to neuron-basal medium (1.5 ml, Gibco) with 2% B27 (Gibco) and replaced every 3–4 days. Treatment with 5-fluoro-5'-deoxyuridine (20 μg/ml, Sigma, St. Louis, MO) on the fourth day after plating was used to block cell division of non-neuronal cells, which helped to stabilize the cell population. The cultures were maintained at 37°C in a 5% CO_2 _humidified atmosphere. Cells were used for electrophysiological recordings 7–23 days after plating.

### Slice preparation

Experiments were performed on 400 μm transverse hippocampal slices from 14- to 17-day-old Sprague-Dawley rats. After decapitation, the brain was removed and placed in oxygenated (95% O_2_/5% CO_2_) artificial cerebrospinal fluid (ACSF) at 4°C. Slices were cut from the dorsal hippocampus with a vibratome (Leika VT 1000S) and maintained at room temperature (23–25°C) in a holding chamber filled with oxygenated ACSF. After an equilibration period of at least 2 h, a single slice was transferred to the recording chamber, where it was continuously perfused with oxygenated ACSF (23–25°C) at a flow rate of 2.5–3 ml/min.

### Expression of recombinant GlyRs

All constructs were expressed in HEK293 cells as previously reported [39]. In brief, HEK293 cells were cultured at 37°C in a humidified atmosphere of 5% CO2 and 95% air. Cells were maintained in DMEM supplemented with 2 mM L-glutamine, 10% fetal bovine serum, and 100 units/ml penicillin/streptomycin (all from Invitrogen). Transient transfection of HEK293 cells was carried out by using the Lipofectamine 2000 reagent (Invitrogen) according to the supplied protocol. Co-transfection with a green fluorescent protein expression vector, pEGFP-N1, was used to enable identification of transfected cells for patch clamping in some experiments. When co-transfecting the GlyR α and β subunits, their respective cDNAs were combined in a ratio of 1:2 to ensure the formation of functional heterooligomers. Taking advantage of the insensitivity of αβ heteromeric GlyR to picrotoxin [64], we further tested the picrotoxin sensitivity to ensure the formation of heterooligomers. After exposure to transfection solution for 6 h, cells were washed twice using the culture medium and used for electrophysiological recordings over following 16–48 h.

### Solutions and drugs

The standard external solution for cultured neurons recording contained (mM): 150 NaCl, 5 KCl, 1 MgCl_2_, 2 CaCl_2_, 10 N-hydroxyethylpiperazine-NV-2-ethanesulphonic acid (HEPES), 10 Glucose (pH 7.4; osmolarity 310–320 mOsm/l). The patch pipette solution for cultured neurons recording was (mM): 120 KCl, 30 NaCl, 1 MgCl_2_, 0.5 CaCl_2_, 5 EGTA, 2 Mg-ATP, 10 HEPES. The internal solution was adjusted to pH 7.2 with Tris-base. The ACSF for slices incubation and recording was composed of (mM): 126 NaCl, 2.5 KCl, 2 MgSO_4_, 1.25 NaH_2_PO_4_, 26 NaHCO_3_, 2 CaCl_2 _and 10 D-glucose, aerated with 95% O_2 _and 5% CO_2 _at a final pH of 7.4. The osmolarity of the ACSF was 289–295 mOsm/l. The ionic composition of the internal solution for slice recording was (mM): 140 CsCl, 1 MgCl_2_, 10 HEPES, 0.1 EGTA, 4 NaCl, 2 MgATP and 0.3 NaGTP, adjusted to pH 7.2. Drugs used in the present experiments were purchased from Sigma. Steroidal agents were initially dissolved as concentrated stock solutions in dimethylsulphoxide (DMSO) and subsequently diluted to the desired concentration in standard external solution. The final concentration of DMSO employed in the experiments was always ≤ 0.1%, which had no detectable effect on *I*_Gly _in vehicle control experiments. Other drugs were first dissolved in ion-free water and then diluted to the final concentrations in the standard external solution or ACSF just prior to use. Unless otherwise indicated, drugs were applied using a rapid application technique termed the 'Y-tube' method throughout the experiments. This system allows a complete exchange of external solution surrounding a neuron within 20 ms [65].

### Electrophysiological recordings and data analysis

Conventional whole-cell patch-clamp recording was performed under voltage-clamp conditions. Patch pipettes were pulled from glass capillaries with an outer diameter of 1.5 mm on a two-stage puller (PP-830, Narishige, Tokyo, Japan). The resistance between the recording electrode filled with pipette solution and the reference electrode was 4–6 MΩ. Membrane currents were measured using a patch-clamp amplifier (Axon 200B, Axon Instruments, Foster City, CA, USA), sampled and analyzed using a Digidata 1320A interface and a personal computer with Clampex and Clampfit software (Version 9.0.1, Axon Instruments). In most experiments, 70–90% series resistance was compensated. To make outside-out patch, after obtaining the whole-cell recording configuration, the patch was excised by carefully withdrawing the patch pipette from the cell [66]. Additionally, in some experiments that drugs were applied in pipette, the current was measured at least 5 minutes after whole-cell configuration was established to ensure cell dialysis [67]. Unless otherwise indicated, the membrane potential was held at -50 mV throughout the experiment. All experiments were carried out at room temperature (22–25°C).

### Membrane capacitance measurements

Whole-cell voltage clamp was used to step membrane potential from -70 to +20 mV. A cesium based pipette solution was used to block voltage-gated potassium channels. The capacitance transient was recorded in HEK293 cell expressing α2-GlyRs in the absence or presence of 10 μM E_2_. According to the calculation method of capacitance provided by the previous study [46], the capacitance of HEK293 cell expressing α2-GlyRs was estimated to be 33.3 ± 2.2 pF and 33.2 ± 2.4 pF in the absence or presence of E_2_, respectively. The membrane capacitance was not changed by E_2_, indicating that the alteration of plasma membrane capacitance was not involved in E_2 _inhibition of GlyRs.

### Data analysis

Clampfit software was used for data analysis. The continuous theoretical curves for concentration-response relationship of glycine in the presence or absence of steroids were drawn according to a modified Michaelis-Menten equation by the method of least-squares (the Newton-Raphson method) after normalizing the amplitude of the response:

*I *= *I*_max _C^h^/(C^h ^+ EC_50 _^h^)

where *I *is the normalized value of the current, *I*_max _the maximal response, C the drug concentration, EC_50 _the concentration which induced the half-maximal response and *h *the apparent Hill coefficient. The curve for the effect of E_2 _on *I*_Gly _was fitted using the following equation:

*I *= *I*_max _(IC_50_)^h^/(C^h ^+ IC_50 _^h^)

where IC_50 _represents the concentration that induced the half-maximal inhibitory effect.

For analysis of the GlyR-mediated tonic currents in HIP slices, all-point histograms of 30 s epochs at period a1, a2 and a3 was used to measure the baseline current in different condition of drug application (see Figure 6A), and a Gaussian distribution was fitted to the histogram at period a1, a2 and a3. The GlyR-mediated tonic current was revealed by strychnine application. Therefore, the difference between the means of the fitted Gaussians at period a1 and a3 represents the GlyR-mediated tonic current in the absence of E_2_. Similarly, the difference between period a2 and a3 represents the GlyR-mediated tonic current in the presence of E_2_.

The statistical comparisons were carried out by using Student's *t*-test for two groups' comparison, and one-way analysis of variance (ANOVA) for multiple comparisons. All data were reported as the mean ± standard error (S.E.M.). *P *and *n *represent the value of significance and the number of neurons, respectively. Statistically significant differences were assumed as *P *< 0.05.

## Competing interests

The authors declare that they have no competing interests.

## Authors' contributions

PJ and YK carried out all electrophysiological experiments and wrote the manuscript. XBZ participated in the electrophysiological experiment in slices and revised the manuscript. WW participated in cell culture. TLX and CFL conceived of the study, and participated in its design and coordination. All authors read and approved the final manuscript.

## References

[B1] Beyer C Estrogen and the developing mammalian brain Anat Embryol (Berl) 1999 199 379 390 10.1007/s004290050236 10221449

[B2] Prange-Kiel J Rune GM Direct and indirect effects of estrogen on rat hippocampus Neuroscience 2006 138 765 772 10.1016/j.neuroscience.2005.05.061 16324798

[B3] Maggi A Ciana P Belcredito S Vegeto E Estrogens in the nervous system: mechanisms and nonreproductive functions Annu Rev Physiol 2004 66 291 313 10.1146/annurev.physiol.66.032802.154945 14977405

[B4] Nadal A Diaz M Valverde MA The estrogen trinity: membrane, cytosolic, and nuclear effects News Physiol Sci 2001 16 251 255 11719599 10.1152/physiologyonline.2001.16.6.251

[B5] Collins P Webb C Estrogen hits the surface Nat Med 1999 5 1130 1131 10.1038/13453 10502813

[B6] Edwards DP Regulation of signal transduction pathways by estrogen and progesterone Annu Rev Physiol 2005 67 335 376 10.1146/annurev.physiol.67.040403.120151 15709962

[B7] Levin ER Cellular functions of plasma membrane estrogen receptors Steroids 2002 67 471 475 10.1016/S0039-128X(01)00179-9 11960623

[B8] Abraham IM Todman MG Korach KS Herbison AE Critical in vivo roles for classical estrogen receptors in rapid estrogen actions on intracellular signaling in mouse brain Endocrinology 2004 145 3055 3061 10.1210/en.2003-1676 14976146

[B9] Qiu J Bosch MA Tobias SC Grandy DK Scanlan TS Ronnekleiv OK Kelly MJ Rapid signaling of estrogen in hypothalamic neurons involves a novel G-protein-coupled estrogen receptor that activates protein kinase C J Neurosci 2003 23 9529 9540 14573532 10.1523/JNEUROSCI.23-29-09529.2003 PMC6740471

[B10] Toran-Allerand CD Guan X MacLusky NJ Horvath TL Diano S Singh M Connolly ES Jr Nethrapalli IS Tinnikov AA ER-X: a novel, plasma membrane-associated, putative estrogen receptor that is regulated during development and after ischemic brain injury J Neurosci 2002 22 8391 8401 12351713 10.1523/JNEUROSCI.22-19-08391.2002 PMC6757764

[B11] Nadal A Ropero AB Fuentes E Soria B The plasma membrane estrogen receptor: nuclear or unclear? Trends Pharmacol Sci 2001 22 597 599 10.1016/S0165-6147(00)01846-0 11730951

[B12] Valverde MA Rojas P Amigo J Cosmelli D Orio P Bahamonde MI Mann GE Vergara C Latorre R Acute activation of Maxi-K channels (hSlo) by estradiol binding to the beta subunit Science 1999 285 1929 1931 10.1126/science.285.5435.1929 10489376

[B13] Greenspan JD Craft RM LeResche L Arendt-Nielsen L Berkley KJ Fillingim RB Gold MS Holdcroft A Lautenbacher S Mayer EA Studying sex and gender differences in pain and analgesia: a consensus report Pain 2007 132 Suppl 1 S26 45 10.1016/j.pain.2007.10.014 17964077 PMC2823483

[B14] Rudick CN Woolley CS Estrogen regulates functional inhibition of hippocampal CA1 pyramidal cells in the adult female rat J Neurosci 2001 21 6532 6543 11517242 10.1523/JNEUROSCI.21-17-06532.2001 PMC6763095

[B15] Rudick CN Gibbs RB Woolley CS A role for the basal forebrain cholinergic system in estrogen-induced disinhibition of hippocampal pyramidal cells J Neurosci 2003 23 4479 4490 12805288 10.1523/JNEUROSCI.23-11-04479.2003 PMC6740774

[B16] Ledoux VA Woolley CS Evidence that disinhibition is associated with a decrease in number of vesicles available for release at inhibitory synapses J Neurosci 2005 25 971 976 10.1523/JNEUROSCI.3489-04.2005 15673678 PMC6725609

[B17] Li W Jin X Covey DF Steinbach JH Neuroactive steroids and human recombinant rho1 GABAC receptors J Pharmacol Exp Ther 2007 323 236 247 10.1124/jpet.107.127365 17636008

[B18] Song W Chattipakorn SC McMahon LL Glycine-gated chloride channels depress synaptic transmission in rat hippocampus J Neurophysiol 2006 95 2366 2379 10.1152/jn.00386.2005 16381810

[B19] Zhang LH Xu L Xu TL Glycine receptor activation regulates short-term plasticity in CA1 area of hippocampal slices of rats Biochem Biophys Res Commun 2006 344 721 726 10.1016/j.bbrc.2006.03.198 16631121

[B20] Zhang LH Gong N Fei D Xu L Xu TL Glycine Uptake Regulates Hippocampal Network Activity via Glycine Receptor-Mediated Tonic Inhibition Neuropsychopharmacology 2008 33 3 701 711 17522628 10.1038/sj.npp.1301449

[B21] Lynch JW Molecular structure and function of the glycine receptor chloride channel Physiol Rev 2004 84 1051 1095 10.1152/physrev.00042.2003 15383648

[B22] Graham BA Schofield PR Sah P Callister RJ Altered inhibitory synaptic transmission in superficial dorsal horn neurones in spastic and oscillator mice J Physiol 2003 551 905 916 10.1113/jphysiol.2003.049064 12837931 PMC2343288

[B23] Li Y Xu TL State-dependent cross-inhibition between anionic GABA(A) and glycine ionotropic receptors in rat hippocampal CA1 neurons Neuroreport 2002 13 223 226 10.1097/00001756-200202110-00010 11893914

[B24] Chattipakorn SC McMahon LL Strychnine-sensitive glycine receptors depress hyperexcitability in rat dentate gyrus J Neurophysiol 2003 89 1339 1342 10.1152/jn.00908.2002 12612034

[B25] Zhang LH Gong N Fei D Xu L Xu TL Glycine uptake regulates hippocampal network activity via glycine receptor-mediated tonic inhibition Neuropsychopharmacology 2008 33 701 711 10.1038/sj.npp.1301449 17522628

[B26] Harvey RJ Depner UB Wassle H Ahmadi S Heindl C Reinold H Smart TG Harvey K Schutz B Abo-Salem OM GlyR alpha3: an essential target for spinal PGE2-mediated inflammatory pain sensitization Science 2004 304 884 887 10.1126/science.1094925 15131310

[B27] Aguayo LG van Zundert B Tapia JC Carrasco MA Alvarez FJ Changes on the properties of glycine receptors during neuronal development Brain Res Brain Res Rev 2004 47 33 45 10.1016/j.brainresrev.2004.06.007 15572161

[B28] Young TL Cepko CL A role for ligand-gated ion channels in rod photoreceptor development Neuron 2004 41 867 879 10.1016/S0896-6273(04)00141-2 15046720

[B29] McDearmid JR Liao M Drapeau P Glycine receptors regulate interneuron differentiation during spinal network development Proc Natl Acad Sci USA 2006 103 9679 9684 10.1073/pnas.0504871103 16763051 PMC1480466

[B30] Rune GM Frotscher M Neurosteroid synthesis in the hippocampus: role in synaptic plasticity Neuroscience 2005 136 833 842 10.1016/j.neuroscience.2005.03.056 16344155

[B31] Amateau SK Alt JJ Stamps CL McCarthy MM Brain estradiol content in newborn rats: sex differences, regional heterogeneity, and possible de novo synthesis by the female telencephalon Endocrinology 2004 145 2906 2917 10.1210/en.2003-1363 14988386

[B32] Chesnoy-Marchais D Meillerais A Oestradiol rapidly enhances spontaneous glycinergic synaptic inhibition of hypoglossal motoneurones J Neuroendocrinol 2008 20 233 244 18047550 10.1111/j.1365-2826.2007.01635.x

[B33] Hu R Cai WQ Wu XG Yang Z Astrocyte-derived estrogen enhances synapse formation and synaptic transmission between cultured neonatal rat cortical neurons Neuroscience 2007 144 1229 1240 10.1016/j.neuroscience.2006.09.056 17184929

[B34] Razandi M Pedram A Greene GL Levin ER Cell membrane and nuclear estrogen receptors (ERs) originate from a single transcript: studies of ERalpha and ERbeta expressed in Chinese hamster ovary cells Mol Endocrinol 1999 13 307 319 10.1210/me.13.2.307 9973260

[B35] Clarke CH Norfleet AM Clarke MS Watson CS Cunningham KA Thomas ML Perimembrane localization of the estrogen receptor alpha protein in neuronal processes of cultured hippocampal neurons Neuroendocrinology 2000 71 34 42 10.1159/000054518 10644897

[B36] Lee DY Chai YG Lee EB Kim KW Nah SY Oh TH Rhim H 17Beta-estradiol inhibits high-voltage-activated calcium channel currents in rat sensory neurons via a non-genomic mechanism Life Sci 2002 70 2047 2059 10.1016/S0024-3205(01)01534-X 12148697

[B37] Qiu J Bosch MA Jamali K Xue C Kelly MJ Ronnekleiv OK Estrogen upregulates T-type calcium channels in the hypothalamus and pituitary J Neurosci 2006 26 11072 11082 10.1523/JNEUROSCI.3229-06.2006 17065449 PMC6674650

[B38] Yevenes GE Peoples RW Tapia JC Parodi J Soto X Olate J Aguayo LG Modulation of glycine-activated ion channel function by G-protein betagamma subunits Nat Neurosci 2003 6 819 824 10.1038/nn1095 12858180

[B39] Jiang P Yang CX Wang YT Xu TL Mechanisms of modulation of pregnanolone on glycinergic response in cultured spinal dorsal horn neurons of rat Neuroscience 2006 141 2041 2050 10.1016/j.neuroscience.2006.05.009 16806717

[B40] Westergren I Nystrom B Hamberger A Nordborg C Johansson BB Concentrations of amino acids in extracellular fluid after opening of the blood-brain barrier by intracarotid infusion of protamine sulfate J Neurochem 1994 62 159 165 8263515 10.1046/j.1471-4159.1994.62010159.x

[B41] Ahmadi S Lippross S Neuhuber WL Zeilhofer HU PGE(2) selectively blocks inhibitory glycinergic neurotransmission onto rat superficial dorsal horn neurons Nat Neurosci 2002 5 34 40 10.1038/nn778 11740501

[B42] Zeilhofer HU The glycinergic control of spinal pain processing Cell Mol Life Sci 2005 62 2027 2035 10.1007/s00018-005-5107-2 15968463 PMC11139092

[B43] Wiesenfeld-Hallin Z Sex differences in pain perception Gend Med 2005 2 137 145 10.1016/S1550-8579(05)80042-7 16290886

[B44] Korenman SG Comparative binding affinity of estrogens and its relation to estrogenic potency Steroids 1969 13 163 177 10.1016/0039-128X(69)90004-X 5773887

[B45] Langosch D Thomas L Betz H Conserved quaternary structure of ligand-gated ion channels: the postsynaptic glycine receptor is a pentamer Proc Natl Acad Sci USA 1988 85 7394 7398 10.1073/pnas.85.19.7394 2459705 PMC282193

[B46] Mennerick S Lamberta M Shu HJ Hogins J Wang C Covey DF Eisenman LN Zorumski CF Effects on membrane capacitance of steroids with antagonist properties at GABAA receptors Biophys J 2008 95 176 185 10.1529/biophysj.107.124768 18339741 PMC2426621

[B47] Plassart-Schiess E Baulieu EE Neurosteroids: recent findings Brain Res Brain Res Rev 2001 37 133 140 10.1016/S0165-0173(01)00113-8 11744081

[B48] Hojo Y Hattori TA Enami T Furukawa A Suzuki K Ishii HT Mukai H Morrison JH Janssen WG Kominami S Adult male rat hippocampus synthesizes estradiol from pregnenolone by cytochromes P45017alpha and P450 aromatase localized in neurons Proc Natl Acad Sci USA 2004 101 865 870 10.1073/pnas.2630225100 14694190 PMC321772

[B49] Remage-Healey L Maidment NT Schlinger BA Forebrain steroid levels fluctuate rapidly during social interactions Nat Neurosci 2008 11 1327 1334 10.1038/nn.2200 18820691 PMC2577388

[B50] Nakhla AM Khan MS Romas NP Rosner W Estradiol causes the rapid accumulation of cAMP in human prostate Proc Natl Acad Sci USA 1994 91 5402 5405 10.1073/pnas.91.12.5402 7515502 PMC44003

[B51] Chattipakorn SC McMahon LL Pharmacological characterization of glycine-gated chloride currents recorded in rat hippocampal slices J Neurophysiol 2002 87 1515 1525 11877523 10.1152/jn.00365.2001

[B52] Thio LL Shanmugam A Isenberg K Yamada K Benzodiazepines block alpha2-containing inhibitory glycine receptors in embryonic mouse hippocampal neurons J Neurophysiol 2003 90 89 99 10.1152/jn.00612.2002 12660352

[B53] Wang DS Mangin JM Moonen G Rigo JM Legendre P Mechanisms for picrotoxin block of alpha2 homomeric glycine receptors J Biol Chem 2006 281 3841 3855 10.1074/jbc.M511022200 16344549

[B54] Flint AC Liu X Kriegstein AR Nonsynaptic glycine receptor activation during early neocortical development Neuron 1998 20 43 53 10.1016/S0896-6273(00)80433-X 9459441

[B55] Bechade C Colin I Kirsch J Betz H Triller A Expression of glycine receptor alpha subunits and gephyrin in cultured spinal neurons Eur J Neurosci 1996 8 429 435 10.1111/j.1460-9568.1996.tb01226.x 8714713

[B56] Laube B Maksay G Schemm R Betz H Modulation of glycine receptor function: a novel approach for therapeutic intervention at inhibitory synapses? Trends Pharmacol Sci 2002 23 519 527 10.1016/S0165-6147(02)02138-7 12413807

[B57] Backstrom T Andersson A Andree L Birzniece V Bixo M Bjorn I Haage D Isaksson M Johansson IM Lindblad C Pathogenesis in menstrual cycle-linked CNS disorders Ann N Y Acad Sci 2003 1007 42 53 10.1196/annals.1286.005 14993039

[B58] Maguire JL Stell BM Rafizadeh M Mody I Ovarian cycle-linked changes in GABA(A) receptors mediating tonic inhibition alter seizure susceptibility and anxiety Nat Neurosci 2005 8 797 804 10.1038/nn1469 15895085

[B59] Handwerker HO Kobal G Psychophysiology of experimentally induced pain Physiol Rev 1993 73 639 671 8332641 10.1152/physrev.1993.73.3.639

[B60] Nakae A Tanaka T Miyake K Hase M Mashimo T Comparing methods of detection and quantitation of RNA editing of rat glycine receptor alpha3 Int J Biol Sci 2008 4 397 405 18974845 10.7150/ijbs.4.397 PMC2575349

[B61] Meier JC Henneberger C Melnick I Racca C Harvey RJ Heinemann U Schmieden V Grantyn R RNA editing produces glycine receptor alpha3(P185L), resulting in high agonist potency Nat Neurosci 2005 8 736 744 10.1038/nn1467 15895087

[B62] Eichler SA Kirischuk S Juttner R Legendre P Lehmann TN Gloveli T Grantyn R Meier JC Glycinergic Tonic Inhibition of Hippocampal Neurons with Depolarising GABAergic Transmission Elicits Histopathological Signs of Temporal Lobe Epilepsy J Cell Mol Med 2008 10.1111/j.1582-4934.2008.00357.x PMC3828897 19210758

[B63] Gao J Duan B Wang DG Deng XH Zhang GY Xu L Xu TL Coupling between NMDA receptor and acid-sensing ion channel contributes to ischemic neuronal death Neuron 2005 48 635 646 10.1016/j.neuron.2005.10.011 16301179

[B64] Pribilla I Takagi T Langosch D Bormann J Betz H The atypical M2 segment of the beta subunit confers picrotoxinin resistance to inhibitory glycine receptor channels EMBO J 1992 11 4305 4311 1385113 10.1002/j.1460-2075.1992.tb05529.x PMC557003

[B65] Murase K Randic M Shirasaki T Nakagawa T Akaike N Serotonin suppresses N-methyl-D-aspartate responses in acutely isolated spinal dorsal horn neurons of the rat Brain Res 1990 525 84 91 10.1016/0006-8993(90)91323-9 2147117

[B66] Hamill OP Marty A Neher E Sakmann B Sigworth FJ Improved patch-clamp techniques for high-resolution current recording from cells and cell-free membrane patches Pflugers Arch 1981 391 85 100 10.1007/BF00656997 6270629

[B67] Li Y Wu LJ Legendre P Xu TL Asymmetric cross-inhibition between GABAA and glycine receptors in rat spinal dorsal horn neurons J Biol Chem 2003 278 38637 38645 10.1074/jbc.M303735200 12885784

